# Association between hyperuricemia and metabolic syndrome in patients suffering from bipolar disorder

**DOI:** 10.1186/s12888-018-1952-z

**Published:** 2018-12-18

**Authors:** Jingxu Chen, Hongmei Chen, Junhui Feng, Ligang Zhang, Juyan Li, Ran Li, Shaoli Wang, Ian Wilson, Alison Jones, Yunlong Tan, Fude Yang, Xu-Feng Huang

**Affiliations:** 1 Beijing Hui-Long-Guan Hospital, Peking University, Beijing, 100096 China; 2 Jining Psychiatric Hospital, Jining, 272051 China; 3 Illawarra Health and Medical Research Institute and School of Medicine, University of Wollongong, Wollongong, NSW 2522 Australia

**Keywords:** Bipolar, Hyperuricemia, Metabolic syndrome, Ageing

## Abstract

**Background:**

Clinical studies have shown that bipolar patients have increased serum uric acid levels. High serum uric acid levels could play a role contributing to high prevalence of metabolic syndrome. Metabolic syndrome is known to increase the risk of developing a number of life threatening diseases including coronary heart disease, hypertension, and type 2 diabetes. This study investigated the association between hyperuricemia and metabolic syndrome and its components in individuals suffering from bipolar disorders.

**Methods:**

This study recruited 318 inpatients suffering from bipolar disorders from Beijing Hui-Long-Guan Hospital in China and 160 healthy subjects from the same region as the controls. We used National Cholesterol Education Program Adult Treatment Panel III Adapted criteria (NCEP ATP-III A) for the diagnosis of metabolic syndrome. Hyperuricemia was determined as serum uric acid level above 420 μmol/L in men and 360 μmol/L in women (N Engl J Med 359(17):1811–1821, 2008).

**Results:**

Among 318 bipolar patients, there was higher prevalence of metabolic syndrome (42.5%) and hyperuricemia (27.7%) than healthy controls (21.9 and 11.9%). Bipolar patients with metabolic syndrome had increased prevalence of hyperuricemia (OR = 3.0, CI95 [1.7–5.4]). Hypertriglyceridemia and larger waist circumference (WC) were associated with hyperunicemia (OR = 1.8, CI95 [1.1–3.1], OR = 1.9, CI95 [1.1–3.4]). Hyperuricemia was associated with metabolic syndrome in bipolar patients (*p* < 0.001) and especially with hypertriglyceridemia (OR = 1.9, CI95 [1.1–3.1] and increased WC (OR = 2.1 [1.2–4.0]). Bipolar patients over 50 years of age and hyperuricemia were highly prone to develop metabolic syndrome (OR = 14.0, CI95 [5.0–39.0]).

**Conclusions:**

Hyperuricemia was highly associated with development of metabolic disorder particularly for aged patients suffering from bipolar disorders. Early prevention of hyperuricemia and metabolic syndrome may lead better life for bipolar patients when they get older.

## Background

Bipolar disorder is a chronic mental illness, which is associated with a potentially devastating long-term impact on the patient’s health, as well as their job, social relationships and families [1–3]. The World Health Organization reported that bipolar disorder was the seventh among severe diseases in the year 2000 [4]. People suffering from bipolar disorders have high mortality, which could result in a 10–20 year shorter lifespan compared to the general population [5]. These patients have a high prevalence of cardiovascular disease to which 35–40% of deaths are attributable, which is associated with metabolic syndrome [6]. Increased ageing population leads to high burden of bipolar disorder patients [2]. Therefore, it is important to investigate possible contributing factors in order to prevent metabolic syndrome, reduce cardiovascular risk, and allocate health care resources to bipolar patients.

Metabolic syndrome is characterized by visceral obesity, altered lipid and glucose profiles and hypertension, all of which are known to increase the risk of developing cardiovascular diseases and type 2 diabetes [7]. Although the causal factors of developing metabolic syndrome in bipolar disorder patients are largely unknown, some contributing factors are proposed. For example, the risk factors may be due to the side effects of an unhealthy lifestyle including excessive food intake and reduced energy expenditure caused by mental illness or drug treatment, or inability to access appropriate health care [8–10].

Uric acid is the final oxidative product in the degradation of purine nucleotides. It has been evidenced by several studies that high serum uric acid levels were as a potential causal factor leading to high prevalence of metabolic syndrome and its components, including central obesity, hypertension, hyperlipidemia, diabetes, and insulin resistance [11–13]. Recently, clinical studies have confirmed that bipolar patients in all phases of the illness, especially manic episode, had increased serum uric acid levels [14–16]. Study also shows that the estimated effect between bipolar disorder and uric acid levels was partially mediated by metabolic abnormalities [17]. Therefore, the hyperuricemia and metabolic syndrome may reciprocally affect each other, which impairs glucose and lipid metabolism. This study investigated the relationship between hyperuricemia and metabolic syndrome and its components in individuals suffering from bipolar disorder.

## Methods

### Subjects

This study was carried out in Beijing Hui-Long-Guan hospital, a city-owned psychiatric hospital, China. A total of 318 patients (male/female = 150/168) were recruited from the inpatients suffering bipolar disorders. The study was conduction from July 2015 to June 2017. Patients were diagnosed as bipolar disorder based on DSM-IV diagnostic criteria for Bipolar Disorder I/II. Patients were referred by their psychiatrists to our research team. Patients were between 18 and 65 years of age and Han ethnic Chinese, agreed to participate in the study, and signed consent form either themselves or the first degree of their relative if the patient’s condition did not allow him or her to do so. The research team evaluated the patients including their disease history, current symptoms, and general conditions. Those patients excluded were: 1) diagnosed with substance abuse within the previous 3 months, 2) pregnant or breast-feeding, and 3) those suffering from a neural degenerative disease including dementia and mental retardation.

One hundred and sixty healthy controls (male/female = 74/86) having no significant differences in age (± 1 year) and gender ratio to the patients were recruited from the same local community through the advertisement in media and pamphlets distributed to local residents (Table 1). We have performed psychiatric examination to all participants. Controls having a history of mental illness or substance abuse were excluded. The protocol of this study was approved by the Human Ethics Committee of Beijing Hui-Long-Guan Hospital and all patients were provided with a written informed consent in accordance with National Health and Medical Research Council Guidelines.

**Table 1:** Socio-demographic and clinical characteristics of bipolar patient and health control groups

Variable	Patient Group (*n* = 318)	Control Group (*n* = 160)	X^2^ /t	*p*
Age (years)	39.2 ± 13.1	38.5 ± 11.3	0.532	0.595
Male, n (%)	150 (47.2)	74 (46.3)	0.006	0.807
Education (years)	11.8 ± 3.5	11.7 ± 3.9	0.481	0.631
Married, n (%)	175 (55.0)	108 (67.5)	6.852	0.009
Smoker, n (%)	86 (27.0)	26 (16.3)	6.913	0.009
BMI(kg/m^2^)	24.8 ± 3.3	23.3 ± 2.6	4.955	< 0.001
Overweight, n (%)	145 (45.6)	48 (30.0)	10.757	0.001
Diastolic BP (mm Hg)	75.2 ± 8.4	73.8 ± 8.1	1.793	0.074
Systolic BP (mm Hg)	114.6 ± 11.1	113.5 ± 11.1	1.046	0.296
Hypertension, n (%)	91 (28.6)	29 (18.1)	6.231	0.013
WC (cm)	87.5 ± 11.1	83.6 ± 8.9	3.844	< 0.001
Increased WC, n (%)	186 (58.5)	53 (33.8)	26.062	< 0.001
HDL-C (mmol/L)	1.2 ± 0.4	1.3 ± 0.4	3.331	0.001
Low HDL-C, n (%)	151 (47.5)	50 (31.3)	11.512	0.001
Triglyceride (mmol/L)	2.4 ± 1.5	1.4 ± 1.1	6.526	< 0.001
Hypertriglyceridemia, n (%)	118 (37.1)	37 (23.1)	9.497	0.002
FBG (mmol/L)	5.0 ± 1.2	4.8 ± 0.7	2.660	0.008
Hyperglycaemia, n (%)	75 (23.6)	22 (13.8)	6.365	0.012
MetS, n (%)	135 (42.5)	35 (21.9)	19.669	< 0.001
UA (mmol/L)	348.5 ± 91.8	300.2 ± 76.6	5.729	< 0.001
Hyperuricemia	88 (27.7)	19 (11.9)	15.290	< 0.001

Data were mean ± SD unless otherwise indicated. BMI: body mass index; *BP* blood pressure, *WC* waist circumference, *HDL-C* high density lipoprotein cholesterol, *FBG* fasting blood glucose, *MetS* metabolic syndrome, *UA* uric acid

### Assessment

The height and weight of all subjects were measured on the same calibrated weight scale standing barefoot with light clothes. Body mass index was calculated for all subjects. Patients with a BMI ≥25 kg/m^2^ were considered overweight [18]. Waist circumference was measured at the mid-point level between the inferior costal margin and superior iliac crest at the time that the subject was at the end of his/her expiration while standing. A standard mercury manometer was used to measure blood pressure with the patient in a supine position. We recorded two readings with 5 min apart for blood pressure recordings. If one of the two blood pressure readings were greater than 130/85 mmHg, we took a third reading 30 min later. We then used the lowest of three readings in the analysis according to a previous study [19]. All subjects underwent venipuncture between 6:00 am and 7:00 am after overnight fasting (~ 12 h), and the blood samples were analyzed on the same day. Serum concentrations of blood glucose, triglycerides, high-density lipoprotein cholesterol, and uric acid were measured using commercial kits (Beckman Coulter, Brea, California, USA) and an OLYMPUS AU 2700 automatic biochemical analyzer.

### The definition of metabolic syndrome and hyperuricemia

The present study used NCEP ATP-III-A for the diagnosis of metabolic syndrome [20]. This is because it has adjusted waist circumference for those of European descent to be more appropriate for Asian waist size [20] and sets a more stringent fasting glucose concentration to 5.6 mmol/L [21]. NCEP-ATP-III-A criteria requires the presence at least three of the following components: 1) increased waist circumference (WC): WC greater than 90 cm in men or 80 cm in women; 2) hypertriglyceridemia: triglycerides greater than 1.70 mmol/L (150 mg/dL); 3) high density lipoprotein cholesterol (HDL-C): HDL-C lower than 1.03 mmol/L (40 mg/dL) in men or 1.29 mmol/L (50 mg/dL) in women; 4) hypertension: blood pressure greater than 130 mmHg systolic or greater than 85 mmHg diastolic; and 5) hyperglycemia: fasting glucose concentration greater than 5.6 mmol/L (100 mg/dL), or diagnosed as type 2 diabetes. In addition, treatment with specific drugs was also considered including triglyceride, cholesterol, and glucose lowering drugs and blood pressure lowering drugs. Hyperuricemia was defined as serum level of uric acid > 420 μmol/L (7.0 mg/dL) for men and > 360 μmol/L (6.0 mg/dL) for women [22, 23].

### Statistical analysis

Statistical analysis was carried out using SPSS 15.0 for Windows (SPSS Inc., Chicago, USA). Demographics, clinical measures, and laboratory values were reported using measures of means and standard deviation. Group difference was compared using t-test for continuous variables having normal distribution and Mann-Whitney test for variables, which were not normal distribution including triglyceride, illness duration and CPZ equivalent dose. Chi-square test was used for categorical variable analyses. Univariate analysis was used to identify significant association between variables and metabolic syndrome. Simple binary logistic regression was used to establish correlation of metabolic syndrome and hyperuricemia as responding variable. Odds ratios and 95% confidence intervals for all independent variables were determined. Sample size calculation was set at an alpha value of 0.05, power 85% based on a previous study [24]. Marital status, illness duration, and illness episode were eliminated as confounding factors. All statistical tests were two-tailed, with alpha level set at 0.05.

## Results

### Demographic and clinical characteristics of subjects

A total of 362 inpatients suffering from bipolar disorders were enrolled in this study. Forty-four patients (12.2%) were excluded since there were no fasting blood and/or anthropometry data such as body weight, height, blood pressure, and waist circumference. Patients (*n* = 318) and control subjects (*n* = 160) had no significant differences in age and gender ratio (*p* > 0.05). Demographic and clinical characteristics of the study population were shown in Table 1.

The bipolar patient group had lower marriage rate (*p* = 0.009) and higher proportion of smokers (*p* < 0.01) than the control group. Patients suffering from bipolar disorders were overweight (*p* = 0.001), high BMI (*p* < 0.001), larger WC (*p* = 0.013), and low serum HDL-C (*p* = 0.001), increased triglyceride (*p* < 0.001), high fasting blood glucose (*p* = 0.002), and increased uric acid (*p* < 0.001) compared to control subjects without bipolar disorder. There were no statistical differences in systolic and diastolic blood pressures between patients and control subjects (both *p* > 0.05).

### Bipolar patients had high prevalence of metabolic syndrome and hyperuricemia

The prevalence of both hyperuricemia and metabolic syndrome was higher in bipolar patients compared to control subjects (27.7% vs. 11.9%, *p* < 0.001 and 42.5% vs. 21.9%, *p* < 0.001). Patients having metabolic syndrome had increased WC (58.5%), hypertriglyceridemia (37.1%), hyperglycemia (23.6%), and hypertension (28.6%) and decreased HDL-C (47.5%) (Table 1). Bipolar patients had high prevalence of hyperuricemia than control subjects (27.7% vs 11.9%, *p* < 0.001).

### Factors associated with metabolic syndrome and hyperuricemia

Hyperuricemia and metabolic syndrome in bipolar patients were associated with overweight, longer duration of the illness, and manic or mixed episodes (all *p* < 0.05). Metabolic syndrome was associated with ageing and uses of mood stabilizer and antipsychotics (all *p* < 0.05) (Table 2). Hyperuricemia was more common in male patients than female patients (*p* < 0.05). Bipolar patients with hyperuricemia had a high prevalence of metabolic syndrome than bipolar patients without hyperuricemia (Table 3). Hyperuricemia was associated with hypertriglyceridemia and hyperglycemia, but not with hypertension, in bipolar patients.

**Table 2:** Characteristics of bipolar patients with metabolic syndrome and hyperuricemia

Variable	Metabolic syndrome			Hyperuricemia		
	Yes (*n* = 135)	No (*n* = 183)	*p*	Yes (*n* = 86)	No (*n* = 232)	*p*
Age (years)	44.1 (12.5)	35.4 (12.6)	< 0.001	40.5 (13.4)	38.6 (13.0)	0.265
Male, n (%)	67 (49.6)	83 (45.4)	0.450	50 (56.8)	100 (43.5)	0.033
Education (years)	11.5 (3.6)	12.10 (3.4)	0.104	11.4 (3.2)	12.0 (3.5)	0.150
Smoker, n (%)	42 (31.1)	44 (24.0)	0.161	26 (29.5)	60 (26.1)	0.534
Married, n (%)	83 (61.5)	92 (50.3)	0.047	47 (53.4)	128 (55.7)	0.719
Illness duration (year)	15.9 (11.3)	9.9 (9.3)	< 0.001	14.25 (10.6)	11.9 (10.6)	0.013
Over weight, n (%)	85 (63.0)	60 (32.8)	< 0.001	52 (59.1)	93 (40.4)	0.003
Illness episode, n (%)			0.012			0.001
Manic/hypomanic	91 (67.4)	104 (56.8)		68 (77.3)	127 (55.2)	
Depressive	31 (23.0)	69 (37.7)		14 (15.9)	86 (37.4)	
Mixed	5(3.7)	1 (0.5)		3 (3.4)	3 (1.3)	
Euthymic	8(5.9)	9(4.9)		3 (3.4)	14 (6.1)	
BPD type II, n (%)	9 (6.7)	12 (6.6)	0.969	6 (6.8)	15 (6.5)	0.924
Antipsychotics use, n (%)	120 (88.9)	135 (73.8)	0.001	72 (81.8)	183 (79.6)	0.652
CPZ equivalents (mg/d)	344.7 (205.9)	297.7 (248.1)	0.030	3602 (209.8)	307.0 (236.1)	0.081
Mood stabilizer use, n (%)	118 (87.4)	144 (78.7)	0.044	74 (84.1)	188 (81.7)	0.662

Data were mean ± SD unless otherwise indicated. *BPD* bipolar disorder, *CPZ* chlorpromazine

**Table 3:** Association between hyperuricemia and metabolic syndrome and its components

Variable	Hyperuricemia		X^2^	*p*
	Yes(n = 86)	No(n = 232)		
MetS, n (%)	55 (62.5)	80 (34.8)	20.015	< 0.001
Increased WC, n (%)	63 (71.6)	123 (53.5)	8.600	0.003
Low HDL-C, n (%)	47 (53.4)	104 (45.2)	1.713	0.191
Hypertriglyceridemia, n (%)	44 (50.0)	74 (32.2)	8.666	0.003
Hypertension, n (%)	30 (34.1)	61 (26.5)	1.785	0.182
Hyperglycaemia, n (%)	29 (33.0)	46 (20.0)	5.927	0.015

*MetS* metabolic syndrome, *WC* waist circumference, *HDL-C* high density lipoprotein cholesterol

### Association between metabolic syndrome and hyperuricemia

**Fig. 1:**
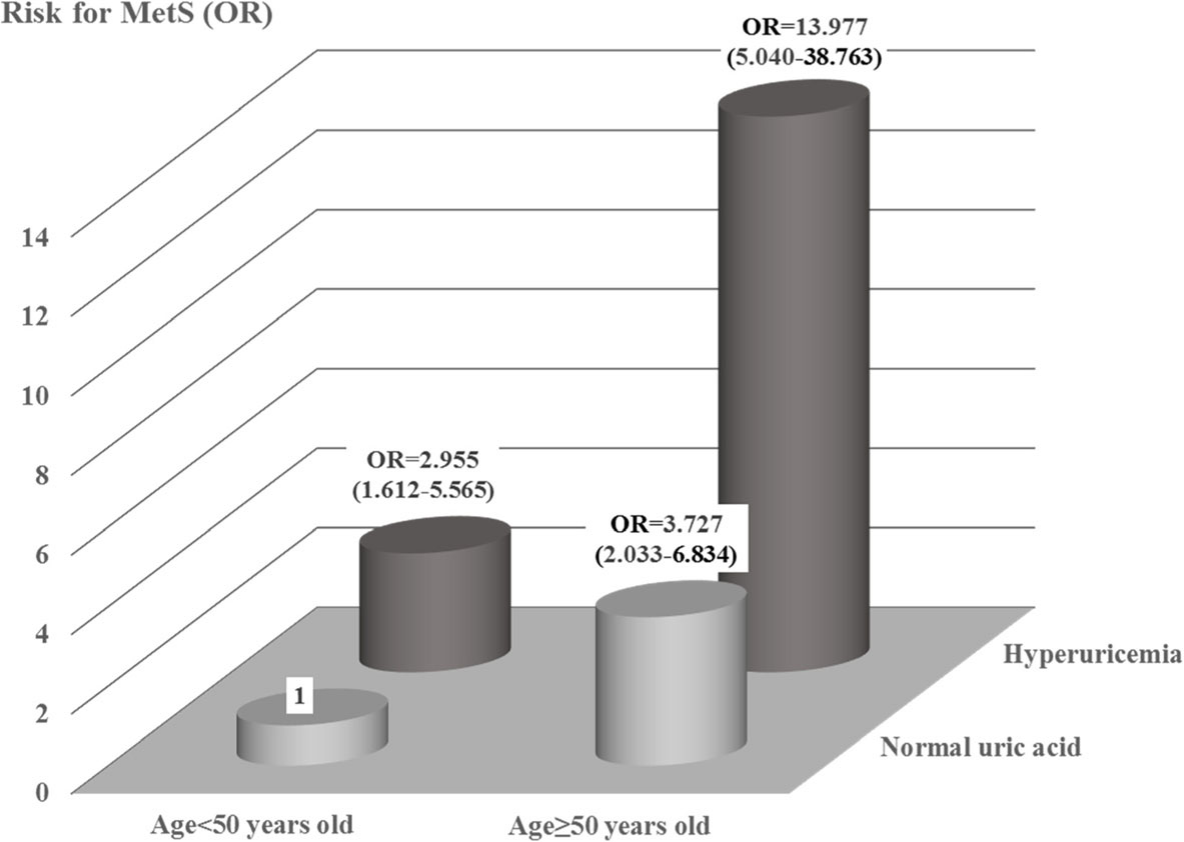
Shows interaction between age ≥ 50 years of age or < 50 years of age and hyperuricemia for metabolic syndrome. Odd ratios are based on 95% confidence interval

The factors that correlated with the presence of metabolic syndrome were ages including 30 and 49 years (OR = 2.2, CI95[1.1–4.2], > 50 years (OR = 8.6, CI95[4.1–18.0], overweight (OR = 3.0, CI95[1.8–5.5]), antipsychotic use (OR = 3.6, CI95[1.7–7.5]), mood stabilizer use (OR = 2.4, CI95[1.1–5.3]) and hyperuricemia (OR = 3.0, CI95[1.7–5.4]) (Table 4). The hyperuricemia were higher with increased WC (OR = 2.1, CI95[1.2–4.0]) and hypertriglyceridemia (OR = 1.9, CI95[1.1–3.1]) (Table 5). Bipolar patients having hyperuricemia and age greater than 50 years were more prone to develop metabolic syndrome (OR = 14.0, CI95[5.0–38.8]) (Fig. 1).

**Table 4:** Associations between the various factors and the risk of having metabolic syndrome

Variable	OR	95%CI	*p*
< 30 years of age	1.000	Reference	–
30–49 years of age	2.149	1.122–4.177	0.021
≥50 years of age	8.587	4.087–18.044	< 0.001
BMI ≥25 kg/m2	3.005	1.767–5.110	< 0.001
Antipsychotics^a^	3.595	1.722–7.497	0.001
Mood stabilizer^b^	2.397	1.087–5.285	0.030
Hyperuricemia^c^	3.029	1.685–5.443	< 0.001

^a:^ use of antipsychotic drug at clinical dose, ^b:^ use of mood stabilizer at clinical dose, ^c:^ serum uric acid > 420 μmol/l for men and > 360 μmol/l for women [21]

**Table 5:** Associations between WC and hypertriglyceridemia with the risk of having hyperuricemia

Variable	OR	95%CI	*p*
Increased WC	2.128	1.233–4.034	0.007
Hypertriglyceridemia	1.870	1.112–3.121	0.017

*WC* waist circumference

## Discussion

The prevalence of metabolic syndrome in bipolar patients varies significantly from 8.5 to 67% from study to study reported in the literature [24–28]. A possible explanation for such large discrepancies may be due to different ethnicities, regions, dietary habits, and genetic backgrounds of patients investigated [29]. For example, high prevalence (67%) was reported in an Australian study and 53% prevalence in an Indian study in bipolar patients suffering metabolic syndrome [25, 27]. Low prevalence of metabolic syndrome in bipolar patients was reported in French (18.5%) and Spanish (22.4%) studies [24, 26]. There are two other Indian studies reports that the prevalence of metabolic syndrome in bipolar patients is about 40%, [20, 30], which is similar to 42% found in this study. On the other hand, the prevalence of metabolic syndrome in control subjects was 21.9% in the present study, which was similar to previous Chinese studies in normal population (24.5%) [31].

Visceral obesity should be closely monitored as the prevalence of larger WC was significantly higher in bipolar patients than control subjects (58.5% vs 33.8%). Visceral obesity is associated with metabolic syndrome, type 2 diabetes and coronary heart disease [32]. The prevalence of metabolic syndrome in bipolar patients was about eight times higher in the bipolar patients with age over 50 years than the bipolar patients with age less than 30 years. This could be due to increased uric acids, which worse insulin sensitivity in elderly patients. Patients having longer illness duration and experiencing manic or mixed episodes had higher rates of metabolic syndrome. The prevalence of gender difference having metabolic syndrome in bipolar patients is controversial. Some studies have reported that the prevalence of metabolic syndrome in male bipolar patients was about twice higher than female patients; but negative and opposite results were also reported in other studies [27, 33, 34]. The data presented in this study suggested no gender differences to develop metabolic syndrome in bipolar patients. About 80% of bipolar patients were prescribed atypical antipsychotic drugs and/or mood stabilizes, which were strongly linked to metabolic syndrome found in the current study.

Our results confirmed previous data that serum uric acid concentrations in bipolar patients were significantly higher than healthy controls [15, 17, 35]. Animal study shows that uric acid induces endothelial dysfunction and overproduction of reactive oxygen species, which has been considered as a major factor of insulin resistance [36]. In humans, hyperuricemia is also associated with insulin resistance and metabolic syndrome [37, 38]. It is possible that elevated uric acid worse insulin sensitivity and metabolic syndrome and vice versa. Both hyperuricemia and metabolic syndrome increase the risk of cardiovascular disease and dementia in elderly people [39]. Ageing is not necessarily associated increased blood uric acid [40]. This study showed that bipolar patient with hyperuricemia and over 50 years of age have very high prevalence for metabolic disorders and its components. Therefore, a special caution should be taken for preventing cardiovascular diseases when there is hyperuricemia in elderly bipolar patients.

Prevention and treatment of hyperuricemia are clearly important in terms of reducing the prevalence of metabolic syndrome in patients suffering from bipolar disorder. Since metabolic syndrome increases the risks of a number of life threatening diseases, reinstalling hyperuricemia back to normal level may help to reduce diabetes and kidney and cardiovascular diseases in these patients.

This study has some limitations. First, only inpatients with bipolar disorder were included, who tend to suffer more severely, have more comorbidities, and higher rates of pharmacological treatment than those of outpatients. Therefore, our data do not necessarily reflect the prevalence of metabolic syndrome of outpatients suffering from bipolar disorder. Second, antidepressant (eg: mirtazapine) uses were not obtained in this study, which could cause weight gain and metabolic disorders [41]. Finally, other possible confounding factors were not ruled out, which could play a role contributing to the development of metabolic syndrome including types and duration of antipsychotic drugs used, diet, physical activity, socioeconomic status, and alcohol use.

## Conclusions

Hyperuricemia is more common in bipolar patients with metabolic syndrome. The high prevalence of metabolic syndrome and hyperuricemia may be due to hyperglycemia, ageing, antipsychotic and mood stabilizer uses, illness duration, and visceral obesity. It is possible that elevated uric acid disrupts metabolic regulation and on the other hand, metabolic disorders could further worse hyperuricemia in bipolar patients. Bipolar patients with hyperuricemia and age greater than 50 years were highly prone to develop metabolic syndrome.

## Abbreviations

BMI: body mass index
BP: blood pressure
BPD: bipolar disorder
CI: confidence interval
CPZ: chlorpromazine
DSM-IV: Diagnostic and Statistical Manual of Mental Disorders, 4th Edition
FBG: fasting blood glucose
HDL-C: high density lipoprotein cholesterol
MetS: metabolic syndrome
NCEP ATP-III A: National Cholesterol Education Program Adult Treatment Panel III Adapted criteria
OR: odds ratio
SPSS: Statistical Package for the Social Sciences
UA: uric acid
WC: waist circumference
